# Supplementary material for: Salinity Treatments Promote the Accumulations of Momilactones and Phenolic Compounds in Germinated Brown Rice
Source: Foods. 2023 Jun 27;12(13):2501. doi: 10.3390/foods12132501 (PMC10340199; doi:10.3390/foods12132501)
Supplement: Supplementary file 1 [file foods-12-02501-s001.zip › foods-2448055-Supplementary.pdf]

foods

MDPI

Variations of Momilactones and Phenolic Compounds in Brown Rice Seeds Germinated under Salinity Treatments

Supplementary Data:

(A)

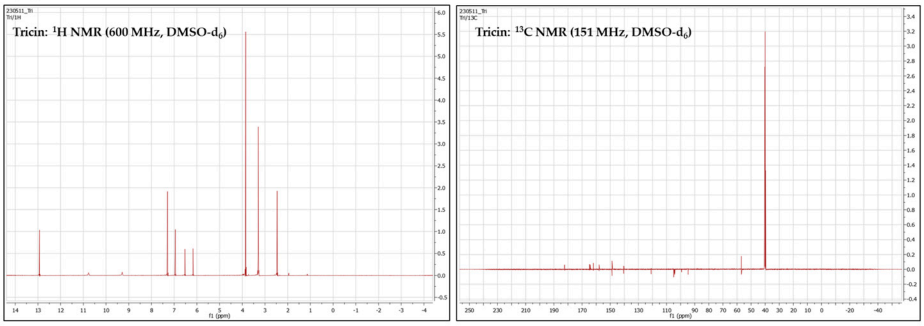

(B)

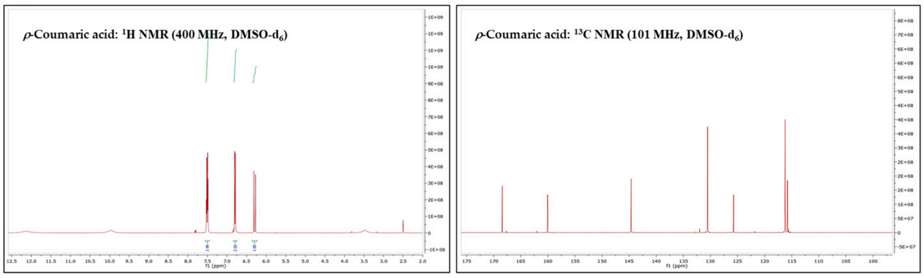

(C)

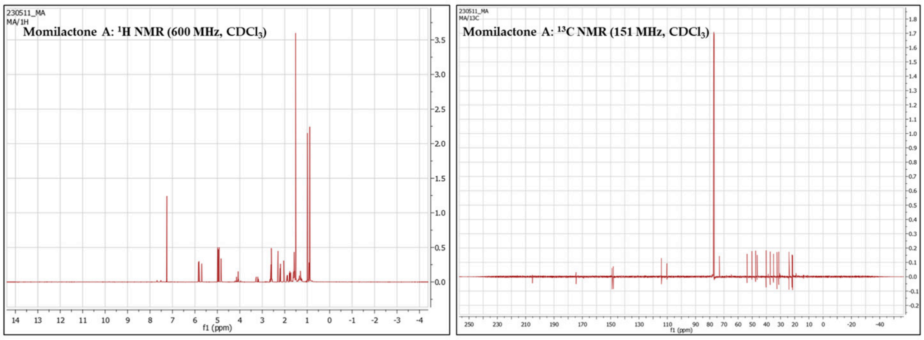

(D)

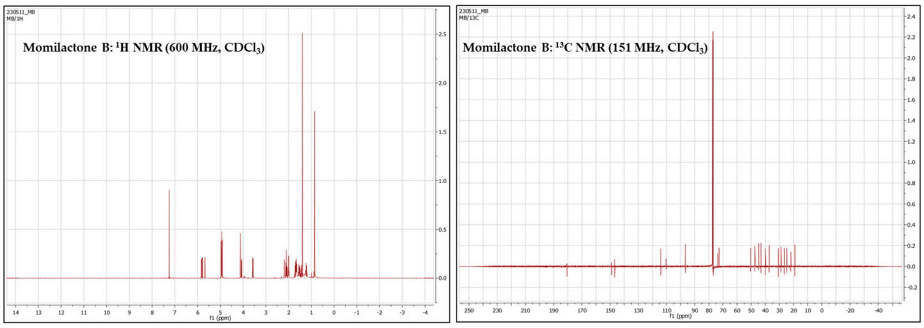

Figure S1. ^{1}H- and ^{13}C-NMR spectrum of isolated **(A)** tricin, **(B)** *p*-coumaric acid, **(C)** momilactone A (MA), and **(D)** momilactone B (MB) in this study.

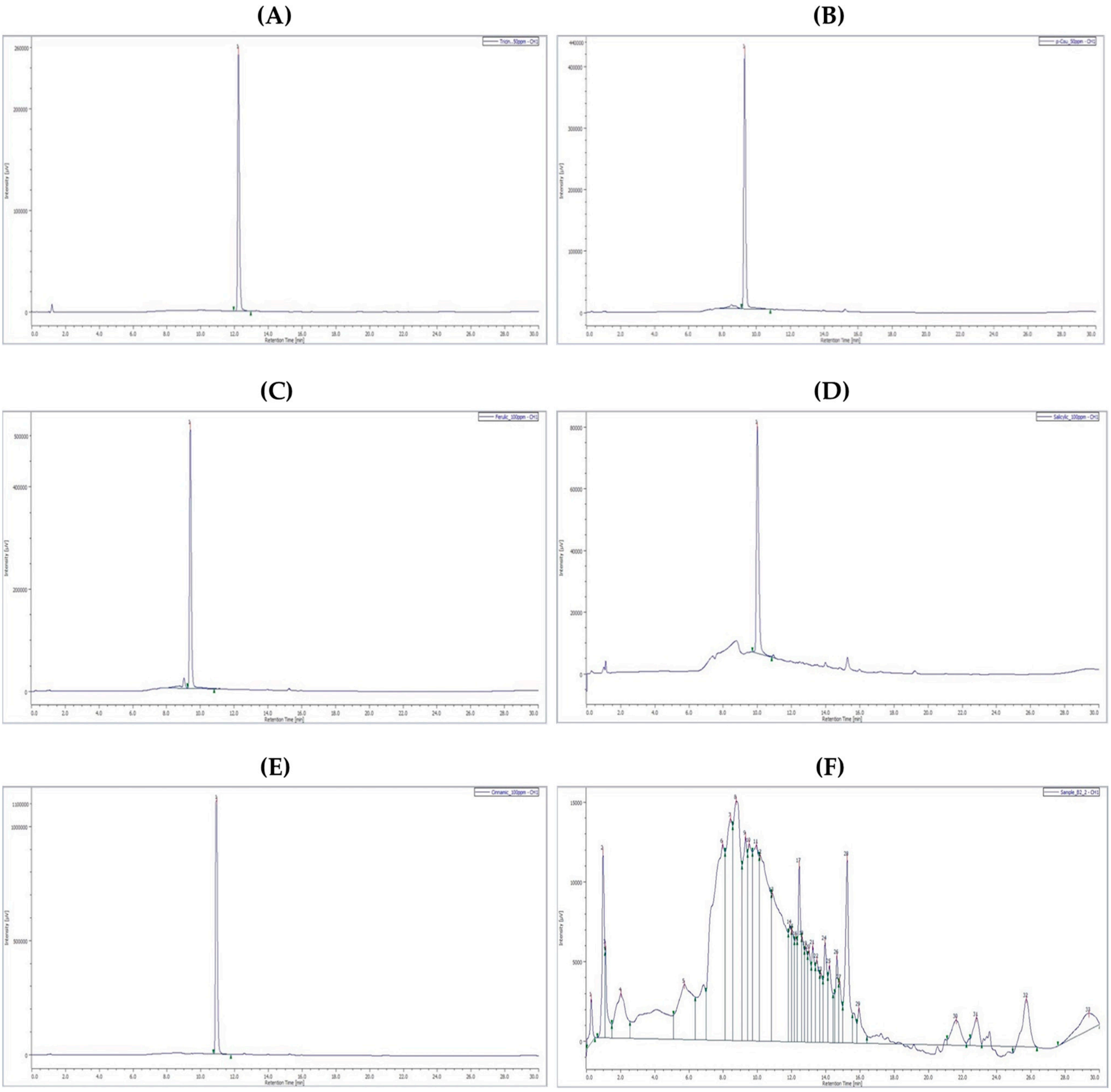

Figure S2. High-performance liquid chromatography (HPLC) chromatograms of standards **(A)** tricin; **(B)** ρ-coumaric acid; **(C)** ferulic acid; **(D)** salicylic acid; **(E)** cinnamic acid, and **(F)** detected phenolic compounds in B2 (75 mM NaCl and 4 day-germination).

(A)

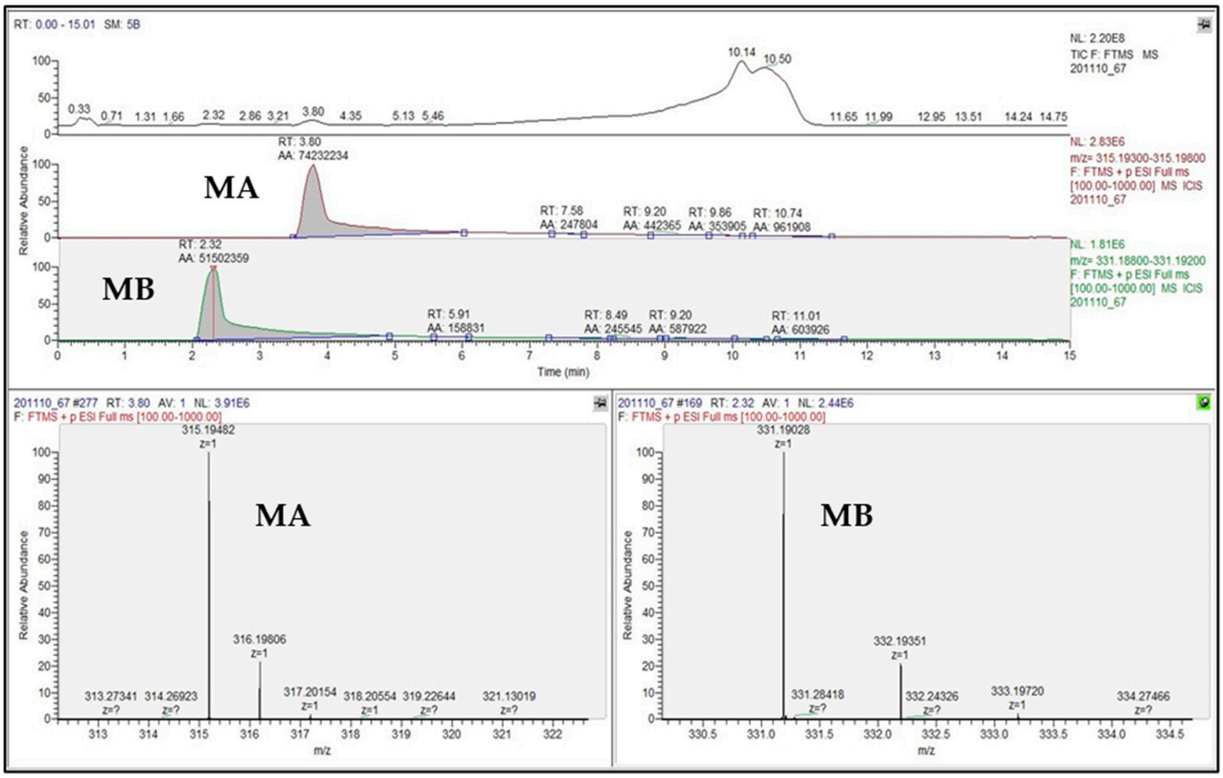

(B)

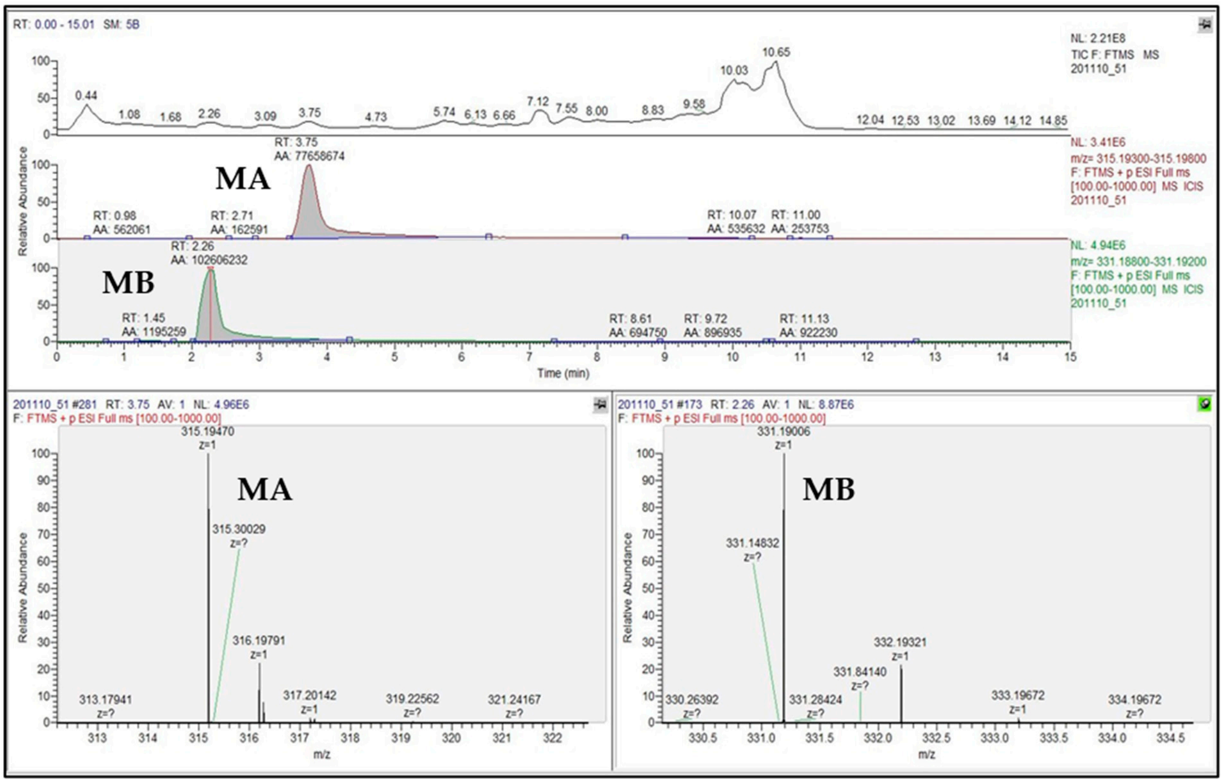

Figure S3. UPLC-ESI-MS chromatograms of **(A)** standard momilactones A (MA) and B (MB), and **(B)** MA and MB detected in GBR in B2 treatment (75 mM NaCl and 4 day-germination).
